# Association between periodontal health status and quality of life: a cross-sectional study

**DOI:** 10.3389/froh.2024.1346814

**Published:** 2024-01-25

**Authors:** Kinan M. Al-Bitar, Jeffrey M. Garcia, Shengtong Han, Arndt Guentsch

**Affiliations:** ^1^ Private Practitioner, Waukesha, WI, United States; ^2^ Department of Surgical and Diagnostic Sciences, Marquette University School of Dentistry, Milwaukee, WI, United States; ^3^ Department of General Dental Sciences, Marquette University School of Dentistry, Milwaukee, WI, United States

**Keywords:** oral health, quality of life, health literacy, periodontitis, periodontal health

## Abstract

**Introduction:**

Attachment loss due to periodontal diseases is associated with functional limitations as well as physical pain and psychological discomfort, which may lead to a reduced quality of life. The purpose of this study is to determine if the oral health status, specifically the periodontal status, influences oral health–related quality of life.

**Materials and methods:**

Survey data were collected in a US dental school clinical setting in a cross-sectional study. Quality of life related to oral health was assessed with the Oral Health Impact Profile-49 (OHIP-49). In addition, DMFT index, periodontal status, and health literacy scores (dental and medical health literacy) were recorded, and the data of *n* = 97 subjects were statistically analyzed.

**Results:**

The DMFT index of the study population was 14.98 ± 6.21 (D: 4.72 ± 4.77; M: 3.19 ± 3.46; F: 7.12 ± 4.62). Of the subjects, 44% were identified as periodontitis cases. These periodontitis cases demonstrated significantly higher OHIP-49 scores (66.93 ± 30.72) than subjects without signs of periodontal diseases (NP) (32.40 ± 19.27, *p* < 0.05). There was also a significant difference between NP patients and patients with gingivitis (66.24 ± 46.12, *p* < 0.05). It was found that there was a statistically significant difference between Stage 3 (severe) periodontitis and periodontal health (*p* = 0.003). Pearson correlations were completed, and positive relationships were found with OHIP-49 and DMFT (0.206, *p* < 0.05), and periodontal risk self-assessment (0.237, *p* < 0.05). Age [odds ratio (OR) 4.46], smoking (OR 2.67), and the presence of mobile teeth (OR 2.96) are associated with periodontitis.

**Conclusions:**

Periodontal diseases may negatively impact the oral health–related quality of life. Patients suffering from periodontitis also showed more missing teeth, which might influence function. Age and smoking are associated with a higher prevalence of periodontitis. A good general health literacy was no guarantee for having an adequate oral literacy.

## 1 Introduction

When patients are asked to evaluate their overall quality of life, it is not uncommon for them to provide an answer based primarily on how they feel from a strictly physical and psychological perspective (1). It is also not uncommon for patients to completely overlook their oral health condition, regardless of its condition, and not attribute their oral health status to their overall quality of life (2). Several studies have found that oral health and overall quality of life tend to go together and that poor oral health conditions have a negative effect on the overall quality of life (3, 4). In addition, it was suggested that oral health problems impair the physical functioning, the social standing, and the wellbeing of individuals, which underlines the association of oral health and general health in terms of impacts on the quality of life (2, 5). The oral health–related quality of life can be evaluated using the Oral Health Impact Profile-49 (OHIP-49) questionnaire (6). The OHIP-49 assesses seven domains, including functional limitation, physical pain, psychological discomfort, physical, psychological, and social disability as well as handicap. The higher the score, the lower the oral health–related quality of life (2).

In the United States alone, much of the population deals with gingivitis and nearly half of the population have periodontitis (7). As we know with periodontitis, the loss of attachment and tooth support leads to discomfort of mobile teeth, further progression of disease, and many times tooth loss. Although replacement of missing teeth is no longer a difficulty with many different available treatment options, it is the destruction of the sites where those teeth once resided and their adjacent conditions that makes the replacement difficult (8). Because of this difficulty, patients are typically put in a position that requires them to manage their predisposing oral health condition, namely, periodontal disease. While gingivitis is reversible and limited to an inflammation of the gingiva, periodontitis is a chronic inflammatory process in which attachment and bone loss occur (9, 10). When bone loss is severe enough, it leads to significant loss of support of the teeth (11). For many patients, this disease process does not happen suddenly and is a result of a lack of awareness and a lack of routine care with their dentist. It has been documented that the strongest risk factor for poor oral health–related quality of life, which was obtained from NHANES (National Health and Nutrition Examination Survey) data, was the perceived need to relieve dental pain (12). While many non-compliant patients would present to their dentist when they experience tooth pain, periodontitis often progresses silently and therefore results in severe damage, which is often too late to address appropriately.

In the unfortunate cases where patients have lost teeth due to periodontal disease or caries, they are subjected to adapting to a new reality. They encounter reduced function, less esthetics, and sometimes comorbidities (13). It is not unknown that there have been associations made between periodontal disease and cardiovascular and mental health (14). Although the connection is very complex and requires more research, correlations are present to provide better answers. For example, diabetes has been shown to have a two-way relationship with periodontal disease. In patients with uncontrolled diabetes, the body's inflammatory process leads to faster and more significant destruction of the periodontium in the presence of bacterial plaque. It is also known that due to the same pathophysiological problems (i.e., RAGE-AGE) with diabetes, wound healing is significantly hindered. The ongoing discomfort and lack of proper healing requires patients to have more frequent visits to their dental provider and longer healing time before addressing other areas of concern (15). With diabetes as an example, it is no surprise that there are several factors that influence quality of life. In addition, numerous chronic systemic diseases are associated with periodontitis, and the prevalence of most chronic diseases increases with age (16). It is suggested that upregulated inflammatory mediators, cytokines, and other pathological reactions are the principal mechanisms linking oral infections to a number of systemic diseases, such as pneumonia, osteoarthritis, rheumatic diseases, inflammatory bowel diseases, kidney diseases, liver diseases, metabolic syndrome and diabetes, cancer, and Alzheimer's disease (17).

The aim of this study was to assess the prevalence of periodontitis in an US-based dental school sample, to identify correlations with the quality of life and health literacy scores, and to determine the oral health literacy of the investigated population. The null-hypothesis is that there is no correlation between the presence of periodontitis and quality of life.

## 2 Methods

### 2.1 Study design

This cross-sectional study was approved by the Institutional Review Board of Marquette University (HR#: 3148). All participants in this study were newly accepted patients at the Marquette University School of Dentistry (Milwaukee, WI) who were scheduled for comprehensive dental examinations. These patients were admitted to the school through initial screening to ensure they qualified as patients and were approached during their radiology appointment prior to their comprehensive examination. The participant would be brought back for clinical examination to confirm periodontal diagnosis. A total of 115 participants were interviewed between 2017 and 2018. Of the 115 analyzed, 97 participants showed comprehensive data for complete analysis.

### 2.2 Inclusion and exclusion criteria

Participants were at least 18 years of age and required to be literate in English. To be included, the participants were required to finish the questionnaire and have a comprehensive evaluation. Participants were excluded if they could not read or write in English. Participants who were evaluated but did not return for clinical examination were also excluded. Further exclusion criteria were mental, vision, or hearing impairments.

### 2.3 Questionnaires

The session involved surveys and questions from Rapid Estimate of Adult Literacy in Dentistry-30 (REALD-30), Short Assessment of Healthy Literacy (SAHL), Periodontal Self-Risk Assessment, OHIP-49, Modified Dental Anxiety Scale, and general demographic information. Three calibrated periodontists (KA, JG, and AG) performed the interviews in a standardized manner.

#### 2.3.1 OHIP-49

The OHIP-49 is a questionnaire that was developed to assess oral health–related quality of life from a patient perspective (6). The questionnaire is comprised of 49 questions with answers on a scale of 0–4. The answers are then tallied for a grand total (maximum of 196). The greater the number, the lower assessed oral health–related quality of life.

#### 2.3.2 Health literacy

##### 2.3.2.1 REALD-30

Oral health literacy was tested using a validated dental word recognition instrument (18). The interviewer provided the participant a sheet of the 30 words. Participants were asked to read each one out loud, and the investigator marked if they were able to correctly read the word. Participants were asked not to guess and say “pass” in the event they needed to guess or did not know the pronunciation of the word. To ensure no non-verbal cues were given, the investigator stood behind the patient for the duration of the questionnaire.

##### 2.3.2.2 SAHL

The SAHL test illustrated medical literacy via word association (19). Participants were given a list of 18 words. Participants were asked to read the words out loud and waited for the investigator to say two words. One of the words was directly related, while the other word was relevant but not as closely associated. The participant was advised to select the word that was most directly related. In the event the participant did not know which option to choose, they were asked to simply state that they did not know. The threshold score was 14, and anything below this score was considered low risk.

#### 2.3.3 Periodontal risk self-assessment

The periodontal risk self-assessment (PRSA) is a 13-item questionnaire, including age, gender, family history of periodontal disease, oral hygiene habits, clinical symptoms of periodontal diseases, smoking habits, and dental history. The answer options are weighted, and scores from 1 to 3 are given. High total scores correlate with the presence of periodontitis (20).

#### 2.3.4 Oral health

All participants were diagnosed based on comprehensive exam information including full mouth radiographs as well as periodontal charting. This exercise was completed by two investigators (AG and KA) independently to ensure consistency. Periodontal status was diagnosed based on the World Workshop of Periodontal Classification (21–23) as periodontal health, gingivitis, and periodontitis. Periodontal health was characterized by <10% of bleeding on probing (BoP), the absence of bone loss, and normal gingival sulcus depths (24). A gingivitis case was diagnosed when BoP scores were ≥10% and the absence of bone loss (25). A periodontitis case was defined as interdental clinical attachment loss (CAL) at ≥2 mm non-adjacent teeth, or buccal or oral CAL ≥3 mm with pocketing >3 mm was detectable at ≥2 teeth (23). The decayed, missing, and filled teeth were recorded as DMFT (26).

### 2.4 Statistical analysis

All data were transferred to a spreadsheet for data organization and analysis. All the variables were described using appropriate statistics. For example, categorical variables were described as frequency and percentage, whereas all continuous variables were described as means and standard deviations. Two-sample *t*-test was used for comparison of other variables (OHIP, DMFT, PRSA, Age, SAHL). The two-sample *t*-test was used for comparison of the OHIP score for two groups (disease and no disease). The one-way ANOVA was used for comparison of the OHIP score of different periodontal stages. Relative risk (RR) and odds ratio (OR) were calculated between healthy and diseased individuals across different covariates. Chi-square test was used to compare SAHL and REALD-30. For all statistical tests, the alpha was set at 0.05 and all statistical analyses were done using statistical software (R version 4.2.2). Using GPower 3.1, a sample size of 97 with effect size of 0.40, the computed achieved power for using ANOVA fixed effect with alpha being 0.05 was 89% (27).

## 3 Results

The demographics and oral health status of the participants are presented in Table 1. Of the included *n* = 97 subjects, *n* = 22 were diagnosed with periodontal health, *n* = 32 with gingivitis, *n* = 21 with stage 1 or 2 periodontitis, and *n* = 22 with stage 3 or 4 periodontitis. The DMFT index of the study population was 14.98 ± 6.21 (D: 4.72 ± 4.77; M: 3.19 ± 3.46; F: 7.12 ± 4.62). The average age of the investigated population was 49 years, with a range from 18 to 84; 56.7% identified as female. When asking about participant race, 62.4% of the participants indicated they were “White” with 19.4% indicating they were “Black.”

**Table 1:** Characteristics of the included subjects based on the presence of periodontitis.

*** ***	Periodontitis (*n* = 43)	Non-periodontitis (*n* = 54)
Age (mean ± SD)	59 ± 14 years	43 ± 18 years
Gender (female)	48%	64%
Body mass index (BMI)	27.8 ± 4.7	29.3 ± 8.3
Decayed teeth	4.4 ± 4.3	4.14 ± 4.8
Missing teeth	3.9 ± 3.6*	2.8 ± 3.4
Filled teeth	7.3 ± 4.6	7.4 ± 4.8
DMFT	15.5 ± 5.6	13.8 ± 6.9

* *p* < 0.05.

### 3.1 Periodontal disease and quality of life

Comparing the OHIP-49 scores of patients with periodontal disease and periodontal health, scores for patients with disease was 63.6, which is significantly higher, compared to the OHIP scores for the patient without disease (35.6; *p* < 0.001).

**Figure 1:**
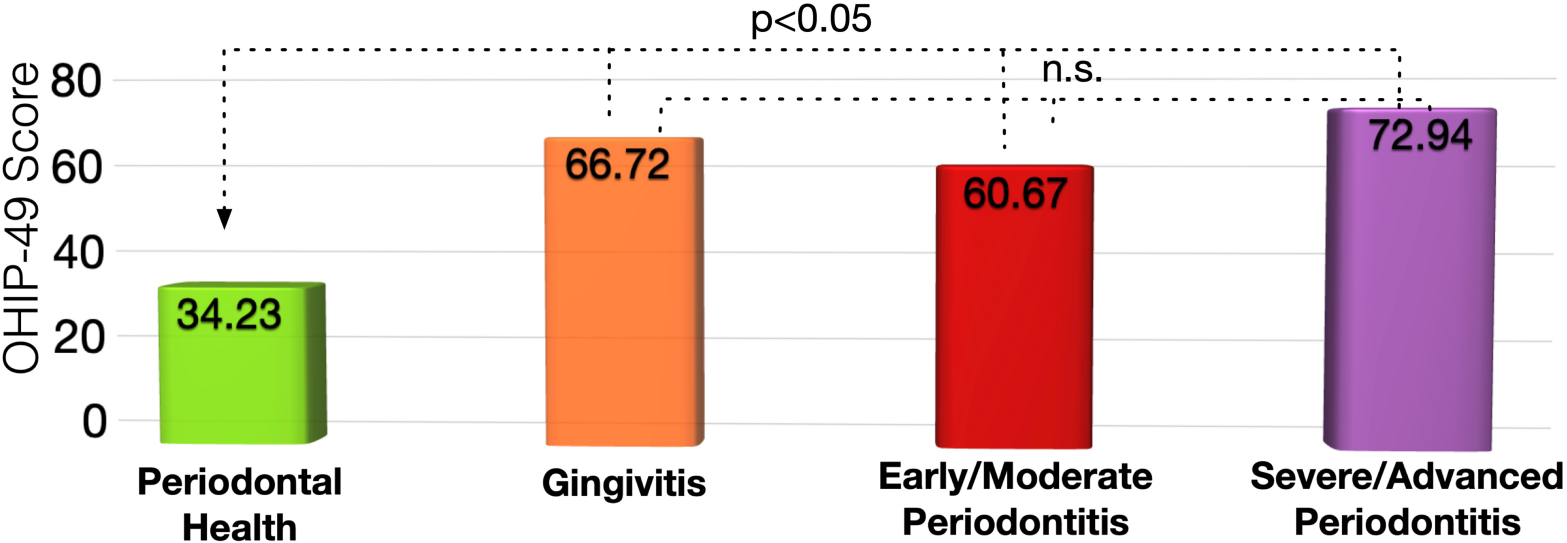
Oral health impact profile-49 scores for patients with different periodontal disease status. The higher the OHIP-49 score, the lower the oral-health related quality of life.

Periodontally healthy patients had an OHIP-49 score of 34.2 ± 20.5, which was significantly lower than for patients with gingivitis with 66.7 ± 47.4 (*p* = 0.015) and patients with stage 3 and 4 periodontitis with 72.9 ± 31.9 (*p* = 0.006). Patients with stage 1 and stage 2 periodontitis had an OHIP-49 score of 60.7 ± 29.0 that was tentatively higher than for patients with periodontal health (*p* = 0.12; Figure 1).

### 3.2 Health literacy

**Figure 2:**
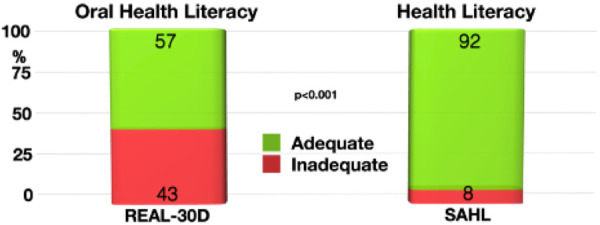
Discrepancy between oral health literacy (REAL-30D) and health literacy (SAHL). Significantly more study participants had adequate health literacy than being literate in oral health aspects.

The subjects of this cohort had a significant discrepancy between health (SAHL) and oral health (REALD-30) literacy (*p* < 0.001). While 92% showed adequate health literacy, only 57% of the participants were found to be having adequate oral health literacy (Figure 2). Patients with inadequate oral health literacy had a higher risk for severe periodontitis (OR: 1.6).

### 3.3 Periodontal self-risk assessment

Patients with periodontal health reported lower PRSA scores (16.2 ± 1.6) than those suffering from periodontal disease (17.6 ± 2.4; *p* < 0.05). However, this was driven by patients with stage 3 and stage 4 periodontitis (19.1 ± 2.3), who had a significantly higher PRSA score than patients with periodontal health (*p* < 0.001). The scores for patients with gingivitis (17.1 ± 2.4) or periodontitis stages 1 and 2 (17.4 ± 2.1) were not significant from those of the participants with periodontal health.

Patient reported that factors such as smoking (*r* = 0.23, *p* < 0.05), having loose teeth (*r* = 0.28, *p* < 0.01), and gingival recession (*r* = 0.20, *p* < 0.05) were significantly correlated with the diagnosis of periodontitis.

Age and smoking were determined as being risk factors for periodontal diseases. While the relative risk for periodontitis was for age 1.28, it was 1.12 for smoking. Relative risks and odds ratios are presented in Table 2.

**Table 2:** Relative risks and odds ratios (95% confidence interval) based on PRSA.

** **	RR	OR
Age	1.28 (0.76–1.85) *p* = 0.51	4.46 (1.32–15.85) *p* = 0.014
Smoking	1.12 (0.85–1.16) *p* = 0.74	2.68 (0.61–18.64) *p* = 0.33
Bleeding gums	1.08 (0.72–1.54) *p* = 0.88	1.7 (0.52–5.51) *p* = 0.39
Mobile teeth	1.12 (0.89–1.17) *p* = 0.84	2.96 (0.46–67.38) *p* = 0.45
Receding gums	1.13 (0.80–1.41) *p* = 0.76	2.44 (0.71–9.54) *p* = 0.24

## 4 Discussion

The patient pool in a dental school typically presents with a specific background in terms of socioeconomic status, education level, and overall health condition. Because of these characteristics, patients may not experience the most ideal oral health status nor the most ideal or controlled overall health quality (28). The study was completed to determine if there was an association between periodontal health and quality of life patients experienced and attempted to identify factors that predispose or highlight possible risk associations. Our findings indicate that, in fact, there are significant associations with periodontal diseases and reduced overall quality of life. Patients with gingivitis and periodontitis had worse overall quality of life scores than those with periodontal health. This is consistent with the existing literature (29).

Especially patients with gingivitis and severe/advanced periodontitis reported a lower oral health–related quality of life. This may indicate that a patient with gingivitis is more aware of the periodontal changes occurring, whereas those in stages 1 and 2 typically are more silent to a patient who has progressed past gingivitis. However, when a patient reaches stage 3, the awareness increases due to possible discomfort of the gingival tissues and teeth as well as mobility of teeth (30). Patients with an advanced stage of periodontitis are also more likely to have general health issues (e.g., diabetes, cardiovascular disease) that may be poorly controlled and contributing to the patient's poor oral health and/or poor overall quality of life (31). The periodontitis risk self-assessment scores were higher in patients with severe/advanced periodontitis, which suggests that patients are typically aware of their condition and may be able to perceive what may be affecting their overall condition as well (32). Combined with the higher OHIP scores in this patient group, this may also indicate that it is at this stage of periodontitis a patient might be aware of their oral health condition and thus is more adept to being self-aware of their overall quality of life. Nisanci Yilmaz et al. reported that the highest OHIP scores and with that lower quality of life were found in patients with stage 4 grade C periodontitis (33). They also found that OHIP scores were significantly related to symptoms of periodontal disease such as bleeding gums, bad odor, and loose or drifting teeth. In a 65-year-old Norwegian population, a researcher found that reduced oral health–related quality came with increased severity of periodontitis (34), which confirmed earlier findings that the severity and progression rate of periodontitis are associated with poor quality of life (35). The good news for those patients is that when they undergo periodontal treatment and participate in a well-structured periodontal maintenance program, the quality of life can be improved and retained (36). This is especially important since the loss of natural teeth due to periodontitis impacts the chewing function, which is associated with diminished nutritional intake (37).

The patients included in this study showed better general health literacy and were less literate in oral health matters. Wehmeyer et al. reported that despite a high level of education of the participants in their cross-sectional study, lower oral health literacy was associated with more severe periodontitis among new and referred patients to their periodontics clinic (38). The present findings suggest that patients with an inadequate REALD-30 score had 1.6 times more severe periodontitis than patients with adequate oral health literacy. Impaired oral health impacts general health and negatively impacts quality of life, and low oral health literacy is associated with reduced quality of life (39). Increasing oral health literacy in educating our patients is critical in addressing poor oral health to prevent oral diseases (40). Nouri and Rudd recommend to use plain language and teach-back by providers as well as the incorporation of oral and aural literacy into community programs and healthcare provider (e.g., dentist, dental assistance, dental hygienists) education and training (41). In the medical field, it is known that the patient awareness of general health concerns is critical in self-care and helping patients seek care when they suspect ailments (42). The same could be said for the dental world, and it may be critical for the dental community to be more of an advocate for the patient to help them self-screen even in cases where symptoms are not as apparent. A possible manner to enhance this is to include our colleagues in medicine to also advocate the oral–systemic connection and educate the patients accordingly. A recent study showed for instance that oral hygiene measures such as brushing teeth are related to the outcome of cardiovascular disease (43). It was also suggested that tooth loss due to periodontal disease or caries caused by oral bacteria impairs the chewing function and health (44), and the disruption of intestinal bacteria can also impair health (45).

Nevertheless, some limitations of this study must be acknowledged. Not all subjects who consented into the study and completed the questionnaires returned for the comprehensive oral exam and were not included in the full analysis. Financial stress, dental anxiety, occupational stress, and perceptions of needs among others might have presented as barriers for patients accessing dental care (46). The selection of questionnaires might also represent a limitation. There are numerous versions of the OHIP questionnaire. John et al. found that the 5-, 14-, 19-, and 49-item versions correlated highly, indicating that these versions measure oral health–related quality of life equally well, with the best being the OHIP-49 (47). However, they also suggest that the OHIP5 is a practical tool for general dentists to assess the oral health–related quality of life, which was also confirmed by others (48). The used oral health literacy tool measures word recognition (18). But there are more ways to assess health literacy (49), including test reading comprehension (50), testing the understanding of medical information (51), or testing numeracy and locate-the-information skills (52). However, several studies found a correlation between the REALD-30 scores and the status of periodontal health (38, 49, 53).The PRSA questionnaire was able to distinguish between periodontal health and periodontitis but failed to discriminate between gingivitis and periodontitis. Other self-reporting tools that are more sophisticated and include questions about systemic health, dietary intake, or psychological stress were shown to be able to assess the individual risk, the need for periodontal treatment, and can differentiate between gingivitis and periodontitis (54). This tool and others such as the periodontal screening score (32) can be helpful screening tools on a population level. In terms of risk or prognosis determination, tooth-level prognostic systems provide better information. Saleh et al. reported recently that the periodontal risk score (PRS), which includes parameters such as age, smoking, diabetes, tooth type, mobility, probing depth, and furcation involvement, was able to predict long-term tooth loss (55).

## 5 Conclusion

Periodontal diseases may negatively impact the oral health–related quality of life. Patients suffering from periodontitis also showed more missing, filled, and decayed teeth, which may have an effect on function and comfort. Age and smoking are associated with a higher prevalence of periodontitis. Good general health literacy was no guarantee for having an adequate oral literacy.

## Data Availability

The raw data supporting the conclusions of this article will be made available by the authors, without undue reservation.
